# The Chaetocerosphere: *Chaetoceros* Species and Associated Prokaryotes in a Coastal Area

**DOI:** 10.1111/1758-2229.70398

**Published:** 2026-09-19

**Authors:** Anna Chiara Trano, Raffaella Casotti, Roberta Piredda

**Affiliations:** ^1^ Department of Integrative Marine Ecology Stazione Zoologica Anton Dohrn Naples Italy; ^2^ Department of Research Infrastructures for Marine Biological Resources Stazione Zoologica Anton Dohrn Naples Italy; ^3^ NBFC, National Biodiversity Future Center Palermo Italy

**Keywords:** 16S rRNA gene, *Chaetoceros*, networks, phycosphere, prokaryotes

## Abstract

The phycosphere, the organic‐rich microenvironment surrounding phytoplankton cells, mediates interactions between algae and other microorganisms, mainly prokaryotes. We investigated associations between *Chaetoceros* spp., one of the most abundant diatom genera in coastal waters, and particle‐attached prokaryotic communities. Monthly samples were collected from October 2017 to September 2018 at a fixed station in the Gulf of Naples (Italy). Light microscopy counts of *Chaetoceros* were integrated with 16S rRNA gene metabarcoding data, and bipartite network analyses were applied to infer putative species‐specific associations. Most inferred associations involved taxa previously reported in diatom‐associated assemblages, such as Alphaproteobacteria and Bacteroidetes and Archaeal taxa including Thermoplasmata and Marine Group II. The networks included both single and (2–3) grouped *Chaetoceros* species. Interestingly, most of the species involved were chain‐forming, although the functional role of this trait in shaping prokaryotic associations remains unclear. Overall, our results broaden the range of prokaryotic taxa potentially associated with *Chaetoceros* and highlight new possible interkingdom interactions in coastal marine ecosystems.

## Graphical abstract

*Chaetoceros* species maintain recurrent distinct prokaryotic assemblages in their phycosphere. Alphaproteobacteria, Bacteroidetes and Archaea Thermoplasmata and Marine Group II are the most represented in the phycosphere. Bipartite networks highlight strong links between the diatom and specific prokaryotic taxa, especially the chain‐forming species.

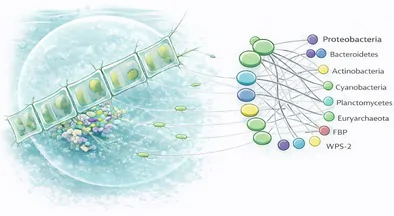

## 1 Introduction

Diatoms (Bacillariophyta) are unicellular photosynthetic eukaryotes representing one of the main lineages of phytoplankton, contributing up to 50% of global primary production in aquatic environments (Falkowski 1994; Field et al. 1998). Despite their ecological success has usually been attributed to their peculiar traits, such as their large central vacuole for nutrient storage, their silica encasing, their advanced biochemical defences, as well as their ecological diversity, several evidence suggest that cross‐kingdom partnership also assist diatoms in surviving in unfavourable environments in the ocean (Amin et al. 2012; Stocker and Seymour 2012). Marine diatoms actively release a variety of organic and inorganic compounds, interacting with and influencing the growth and functioning of prokaryotes through chemical exchanges into a diffusive region surrounding diatom cells, known as the phycosphere (Bell and Mitchell 1972; Seymour et al. 2017). On the other side, prokaryotes have developed mechanisms such as motility and chemotaxis, as well as cell‐surface attachment mechanisms, to maintain close associations within the phycosphere (Stocker and Seymour 2012; Barak‐Gavish et al. 2023), supporting diverse biotic interactions, ranging from synergistic to antagonistic (Vincent and Bowler 2022; Branscombe et al. 2024). The most common prokaryotes observed in the phycosphere of marine diatoms and other phytoplankton belong to Proteobacteria (i.e., *Roseobacter* and *Sulfitobacter*) and Bacteroidetes (i.e., *Cytophaga* and *Flavobacterium*) (Amin et al. 2012; Natrah et al. 2014). In culture, these have been proven to provide diatom cells with vitamin B12, bioavailable iron and even nitrogen (nitrogen‐fixing cyanobacteria) (Amin et al. 2012; Foster et al. 2011). In return, diatoms provide dissolved organic carbon as released in the phycosphere. Generalisation is hampered, though, by the fact that some taxa from the same phyla are able to produce algicidal compounds which may kill diatoms (Lau et al. 2007), and, in turn, some diatoms can produce antibacterial compounds as a defence feature (Desbois et al. 2009). To add complexity, different diatom species or the same species in different physiological states, form distinctive niches for distinct prokaryote associations, while some prokaryotes are able to switch the kind of interaction with diatoms from coexistence to pathogenicity, depending on the environmental conditions (Deng et al. 2022). Indeed, during blooms at sea, usually beneficial prokaryotes have been observed to turn into pathogenic, producing algicide compounds that lyse phytoplankton at the end of blooms, causing the release of organic carbon to the environment (Abate et al. 2024).

Beyond the relevance for ecosystem functioning, the natural or synthetic algae‐prokaryote consortia hold interest for applications in the marine biotechnology field, for instance in wastewater remediation, aquaculture production, greenhouse gas mitigation, biomass and biofuel production (Leong et al. 2019; Mohsenpour et al. 2021), in biorefineries‐cultivation systems (Ramanan et al. 2016) and microalgal harvesting (Gärdes et al. 2011).

*Chaetoceros* is a cosmopolitan species‐rich diatom genus (De Luca et al. 2019; Nef et al. 2022), occupying diverse ecological niches (De Luca et al. 2019). It includes species able to form chains (Li et al. 2017), producing toxins and forming resting stages (Pelusi et al. 2020). Taxonomically well‐studied, with 228 described species (Guiry and Guiry 2018), *Chaetoceros* has been found associate with several microorganisms including epibiotic prokaryotes (Crenn et al. 2018), nitrogen‐fixing cyanobacteria (Foster et al. 2011), tintinnid ciliates (Gómez 2020) and other plankton organisms (Vincent and Bowler 2020).

Despite its relevance, diatoms‐prokaryote interactions are difficult to study, both in the field and in the laboratory. In culture, prokaryotic communities associated with diatoms can be studied by strain isolation or other approaches (e.g., laser scanning microscopy, stable isotope analysis). However, High‐Throughput Sequencing (HTS) is a powerful tool to detail both host and associated prokaryotes both in culture experiments and in natural samples also during blooms (Needham and Fuhrman 2016; Stenow et al. 2023, 2024). However, despite the widespread adoption of HTS, light microscopy (LM) remains a robust and traditionally established method for the identification of diatoms and continues to be widely used due to its reliable morphological resolution, which is not yet fully matched by HTS approaches.

In terms of data analyses, processes‐structuring interactions, co‐existence and dependency among the cross‐kingdom taxa introduce multidimensionality and therefore higher complexity. This can be solved using network analysis, which, despite some difficulties in the interpretation of metrics and proprieties, has been recognised as a powerful tool for the discovery of dynamic microbial interactions (Kajihara and Hynson 2024). In particular, the use of bipartite network analysis has shown to be useful for the exploration of the interactions between nodes belonging to different guilds (e.g., the two microbial kingdoms), as opposite to unipartite global networks where all nodes (species) are categorised equally (Moll et al. 2021).

The aim of this study is to describe the associations of *Chaetoceros* species with prokaryotes at a coastal station of the Gulf of Naples (Italy) for 1 year, with a focus on the spring algal bloom of 2018. To this end, HTS of 16S rRNA gene was used to taxonomically identify the prokaryotes (often up to the genus level), while LM was used to identify the *Chaetoceros* species. The two datasets were then analysed using bipartite network to infer putative associations. The specific aim is to explore the possible role of *Chaetoceros* in microbiome recruitment and the influence of resident environmental prokaryotes in shaping diatom‐prokaryotic associations. The insights from this study will support a better understand of ecological and functional cross‐kingdom consortia for an ecologically important diatom genus.

## 2 Materials and Methods

### 2.1 Study Area

**FIGURE 1:**
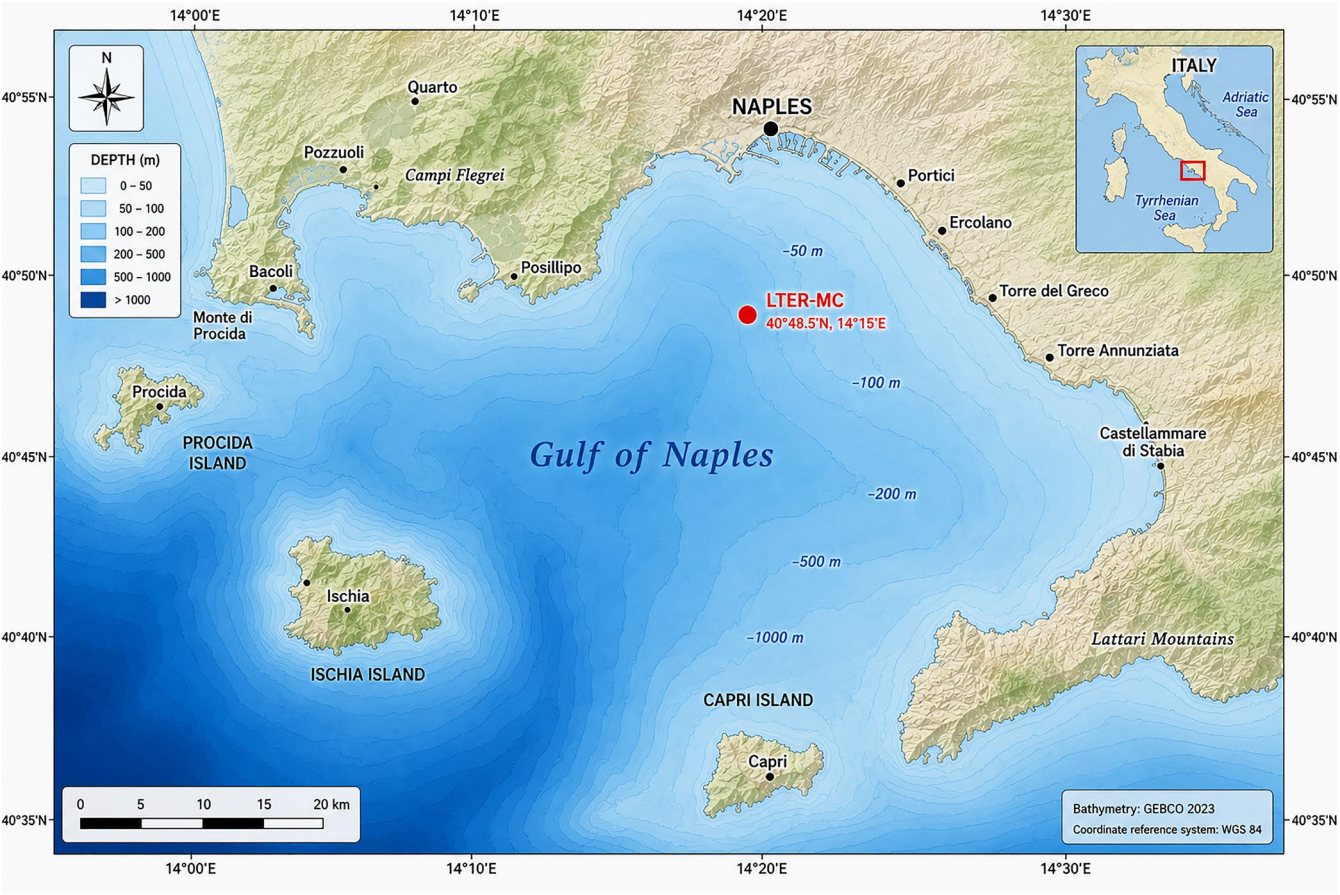
Map of the Gulf of Naples (Italy) with the location of the station LTER‐MC.

The Long‐Term Ecological Research Station MareChiara (LTER‐MC, 40°48.5′N, 14°15′ E) is situated in the Gulf of Naples (Italy), two nautical miles offshore and it lies at the interface between the mesotrophic coastal area and the oligotrophic waters of the mid Tyrrhenian Sea (Cianelli et al. 2017), being alternatively influenced by both (Figure 1).

A strong seasonality characterises the site, with relatively high yearly average chlorophyll *a* concentration (1.72 ± 1.94 mg m^−3^). Total phytoplankton at surface waters show a gradual increase from late February–March and a peak in May (Zingone et al. 2023).

### 2.2 Sample Collection

A total of 15 phytoplankton samples were collected monthly at surface from October 2017 to September 2018, except for June 2018, using Niskin bottles. During the spring phytoplankton bloom (April–May 2018), sampling frequency was increased to every 10 days.

At each sampling event, temperature, salinity, depth, dissolved oxygen concentration and fluorescence profiles were acquired with a SBE 19plus (Sea‐Bird electronic) CTD. For chlorophyll *a* (Chl *a*) concentrations, 200–540 mL of seawater were filtered onto GF/F filters and immediately stored in liquid nitrogen until the analysis. Chl *a* and phaeopigments (Phaeo) were analysed according to Holm‐Hansen et al. (1965) with a Shimadzu RF‐5301 PC spectrofluorometer, daily calibrated with a Chl *a* standard solution (from *Anacystis nidulans* ; Sigma).

Phytoplankton samples (500 mL) for LM counts were disposed in dark glass bottles, fixed with buffered 37% formaldehyde (1.6% final concentration) and stored in the dark at room temperature (RT). Each sample was observed and counted using a Zeiss Axiovert 200 LM (Carl Zeiss, Germany) equipped with a 40× objective (400× total magnification), according to Utermöhl method (Utermöhl 1958). When possible, identification was made at species level, but cells not identifiable were grouped as *Chaetoceros* spp. Samples for DNA extraction and successive sequencing were collected with a 20 μm mesh‐size phytoplankton net towed horizontally at surface. 20 mL of these concentrated phytoplankton net samples were filtered onto 3.0 μm filters (47 mm PC WHA10418312 Whatman‐Nuclepore Track‐Etched Membranes) to retain particle‐attached prokaryotes (mainly to diatoms), using a Swinnex filter holder. All filters were transferred into cryovials, frozen in liquid nitrogen and stored at −80°C until subsequent DNA extraction.

### 2.3 DNA Extractions and Sequencing

DNA extraction was performed using the Qiagen DNeasy Blood and Tissue Kit, according to manufacturer's instructions.

DNA quantity and quality were assessed before PCR amplification using Nanodrop spectrophotometer (ND‐1000UV‐Vis, ThermoFisher, UK). All samples yielded DNA of sufficient quality and concentration for downstream amplification and Illumina MiSeq sequencing. DNA concentrations ranged from 15 to 42 ng μL^−1^, with A260/280 ratios between 1.8 and 1.9.

The extracted DNA samples were sent for PCR amplification and sequenced on an Illumina MiSeq platform (2 × 300 bp paired‐end reads) at the Integrated Microbiome Resource Sequencing Facility at Dalhousie University (Canada). Raw sequences were deposited in the Sequence Read Archive (SRA) of the National Center for Biotechnology Information (NCBI) under BioProject #PRJNA1338595.

The V4‐V5 hypervariable region of the 16S rRNA gene was amplified using the V4‐V5 primer set 515F‐Y (5′ GTGYCAGCMGCCGCGGTAA) and 926R (5′CCGYCAATTYMTTTRAGTTT) (Parada et al. 2016). Preprocessing of raw data was performed by the Bioinformatics Service (BioInforma) of the Stazione Zoologica Anton Dohrn (Naples, Italy). All the libraries were cleaned using Trimmomatic (Bolger et al. 2014), setting the minimum quality per base at 20 phread score and minimum length of the read after cleaning at 250 bp. After primer removal, raw sequencing reads were submitted to the QIIME2 pipeline (version 2025.7) (Bolyen et al. 2019). Amplicon sequence variants (ASVs) were generated through the DADA2 algorithm within QIIME2 (Callahan et al. 2016), with default parameters. The taxonomic assignment of ASVs, was performed against the SILVA database v138.2 (Quast et al. 2012) using a Naïve‐Bayes classifier (Wang et al. 2007); ASVs assigned to chloroplast, mitochondria or ‘unknown’ (i.e., that could not be classified at the kingdom level) were removed and excluded from analyses.

### 2.4 Data Analysis

The prokaryotic ASVs table was rarefied by random subsampling at the lowest number of reads present in all samples (3089 reads) and analyses with the phyloseq (version 1.54.0) (McMurdie and Holmes 2013) and ggplot2 (Wickham 2009) R packages, within R‐software (version 4.5.0). To explore the relationships between environmental data (temperature, salinity and chlorophyll) and ASV (either total ASVs dataset or ASVs selected by bipartite network), the BIO‐ENV analysis (Clarke and Ainsworth 1993) was performed using the Bray–Curtis dissimilarity matrix. Within a set of environmental variables, BIO‐ENV allows the identification of a subset of variables that shows the highest explanatory values.

Bipartite network was generated using the CoNet app (Faust and Raes 2016) within Cytoscape software (version 3.8.0) (Shannon et al. 2003; Dormann et al. 2008) following the pipeline provided by the authors. Network inference algorithm in CoNet combines multiple measures exploiting the fact that different methods make different mistakes. Thus, edges predicted by one method that are not supported by the others will be filtered out reducing the number of false positives.

Briefly, from the Methods menu of Cytoscape, we selected Pearson, Spearman and Bray Curtis as measures of inferences and the threshold of 1000 as the value of the edge number selection included for each measure. Despite the data could not be normally distributed, in the CoNet framework multiple association measures (Pearson, Spearman and Bray–Curtis) are combined to increase robustness, as each method captures different types of relationships and reduces method‐specific biases. Edges are retained only when supported by multiple metrics, thereby minimising false positives (Faust and Raes 2016). This step produced an initial multigraph, where nodes (taxa) are connected by up to three different measure‐specific edges. Then, assessment of edge significance was performed in two steps: in step 1 we computed *p* values for each of the three correlation measures selected (Pearson, Spearman and Bray Curtis) by permutation distributions (selecting shuffle_rows and edgeScores in the Randomisation menu). In step 2, a *p* values merging and bootstrap calculation was performed for each edge (in the Randomisation menu we loaded permutation file generated in step 1; selected brown as *p* value merging method and bootstrap with 100 iterations as resampling method). Using the permutation and bootstrap distributions computed, the final network was generated. In the preprocessing and filter menu we selected positive edges only and relationships were inferred only between different object types (bipartite network). Bipartite graph is a particular form of network used to explore interactions between two distinct classes of objects (partitions), such as plant‐pollinator, parasite–host or prey–predator and the output includes only the connections between nodes from the two partitions whereas the nodes within the same partition are not allowed to be connected to one another. In our analysis, two disjoint datasets were used as input: (i) prokaryotic dataset normalised including ASVs with total abundances ≥ 5 reads; (ii) relative abundance table of cell counts for all the *Chaetoceros* species. Results were visualised in Cytoscape and summarised using the Circos online tool (mkweb.bcgsc.ca/tableviewer) (Krzywinski et al. 2009).

## 3 Results

### 3.1 *Chaetocero*s spp.

**FIGURE 2:**
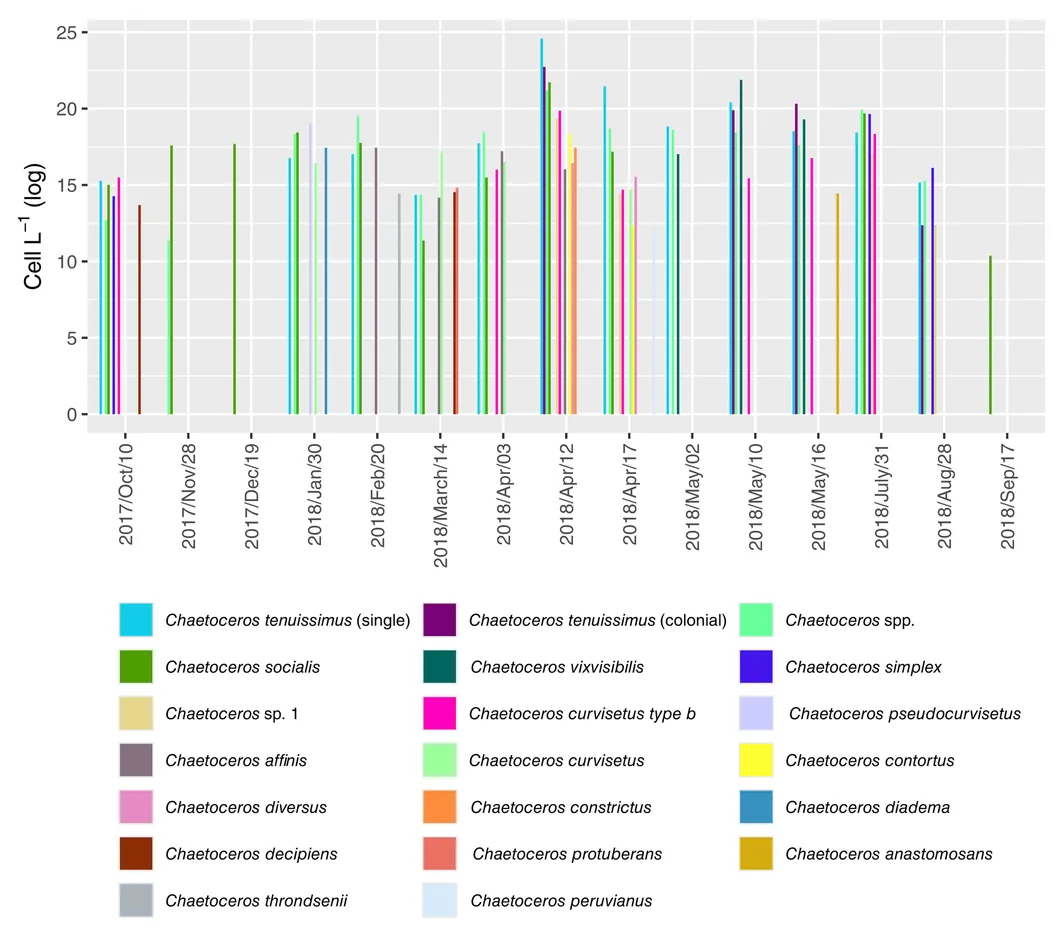
*Chaetoceros* species abundance (cell L^−1^) at the LTER‐MC station during the sampling period.

Diatoms were always the most abundant phytoplankton group in the samples, and *Chaetoceros* spp. were the most represented diatoms at almost all sampling dates. Being significantly represented in the LTER‐MC phytoplankton time series, especially during the spring bloom (with abundance > 90% between April and May), successive analyses have concentrated on this species. From the counts, a total of 20 *Chaetoceros* were identified during the year (Data S1), all at the species level, except for *C*. spp. which includes all the unassigned *Chaetoceros*. Most *Chaetoceros* species concentrations peaked on April 12, up to a total of 2.0 × 10^7^ cell L^−1^, with *Chaetoceros tenuissimus* (solitary form) representing 63% of the total. On April 17 this same species (and form) represented 80%, even though total *Chaetoceros* were one order of magnitute less concentrated. *Camponotus decipiens* , *Coronula diadema* , *Chaetoceros pseudocurvisetus* , *Chimarra protuberans* and *Chaetoceros throndseni* were only present in late winter (January/March), while *Chaetoceros simplex* was present in late summer (October 2017 and July/August 2018). *Chaetoceros socialis* had a secondary peak in July (Figure 2).

### 3.2 Prokaryotic Community Composition

A total of 87,112 raw reads were obtained from sequencing, corresponding to 623 ASVs which, after normalisation, resulted into 605 ASVs representing 46,335 reads (Data S2).

**FIGURE 3:**
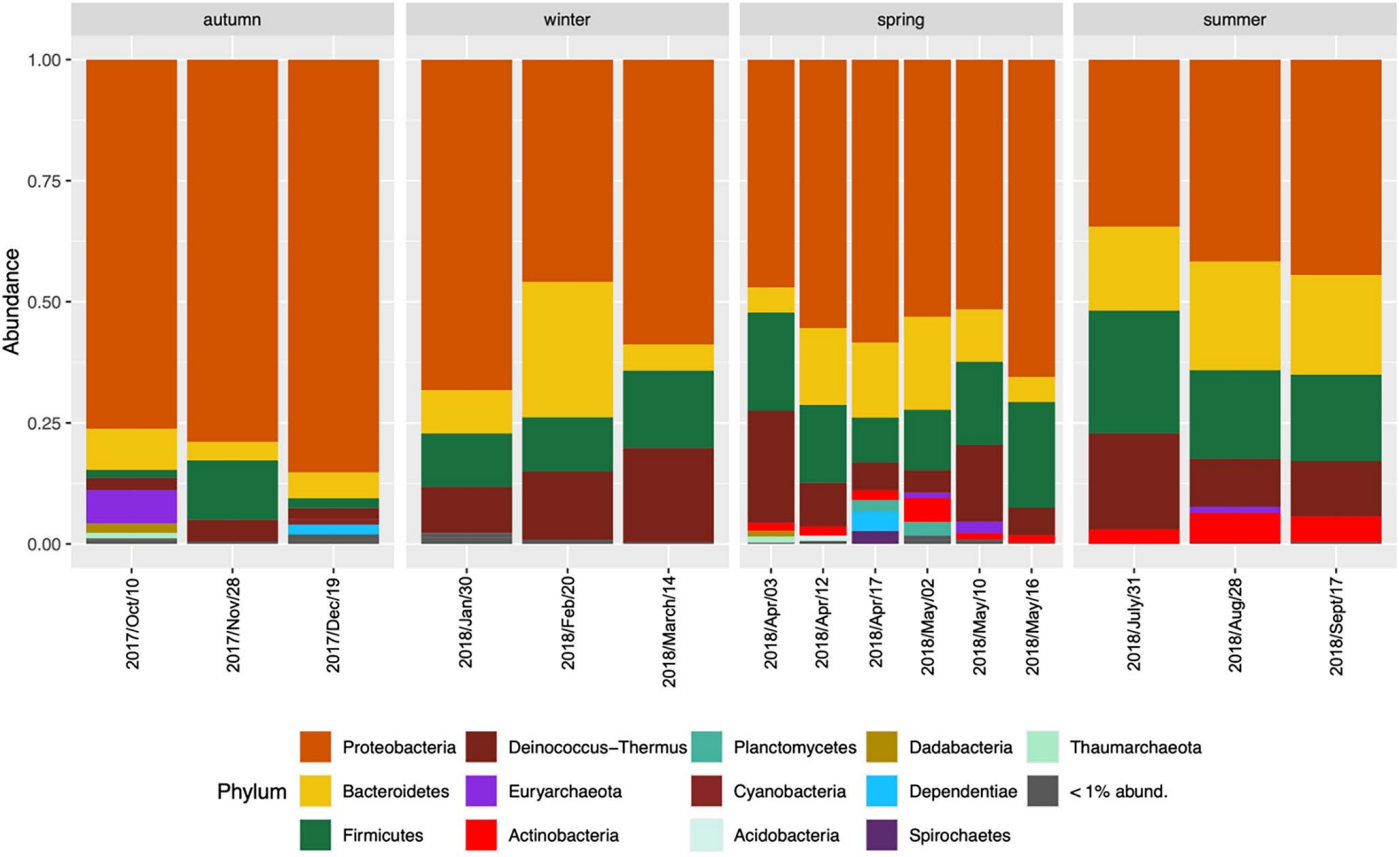
Relative abundances of prokaryotic phyla. The phyla with abundance < 1% in each sample are grouped and represented as ‘< 1% abund.’, in grey.

At the phylum level, the total dataset was dominated by Proteobacteria, Firmicutes, Bacteroidetes and Deinococcus‐Thermus representing 57%, 14% and 13% and 11% of the total, respectively. In the autumn, the community was dominated by Proteobacteria with an average of 80% of total ASVs. In winter and spring, these stayed always > 50%, but Bacteroidetes, Firmicutes and Deinococcus‐Thermus were also contributing to the total (with 14%, 13% and 14% in winter and 16%, 12% and 11% in spring, respectively). In the summer, the contribution of Proteobacteria was the lowest (40%), and the one of Bacteroidetes, Firmicutes and Deinococcus‐Thermus the highest. Actinobacteria appeared in the spring and significantly increased in the summer being completely absent in autumn and winter (Figure 3).

**FIGURE 4:**
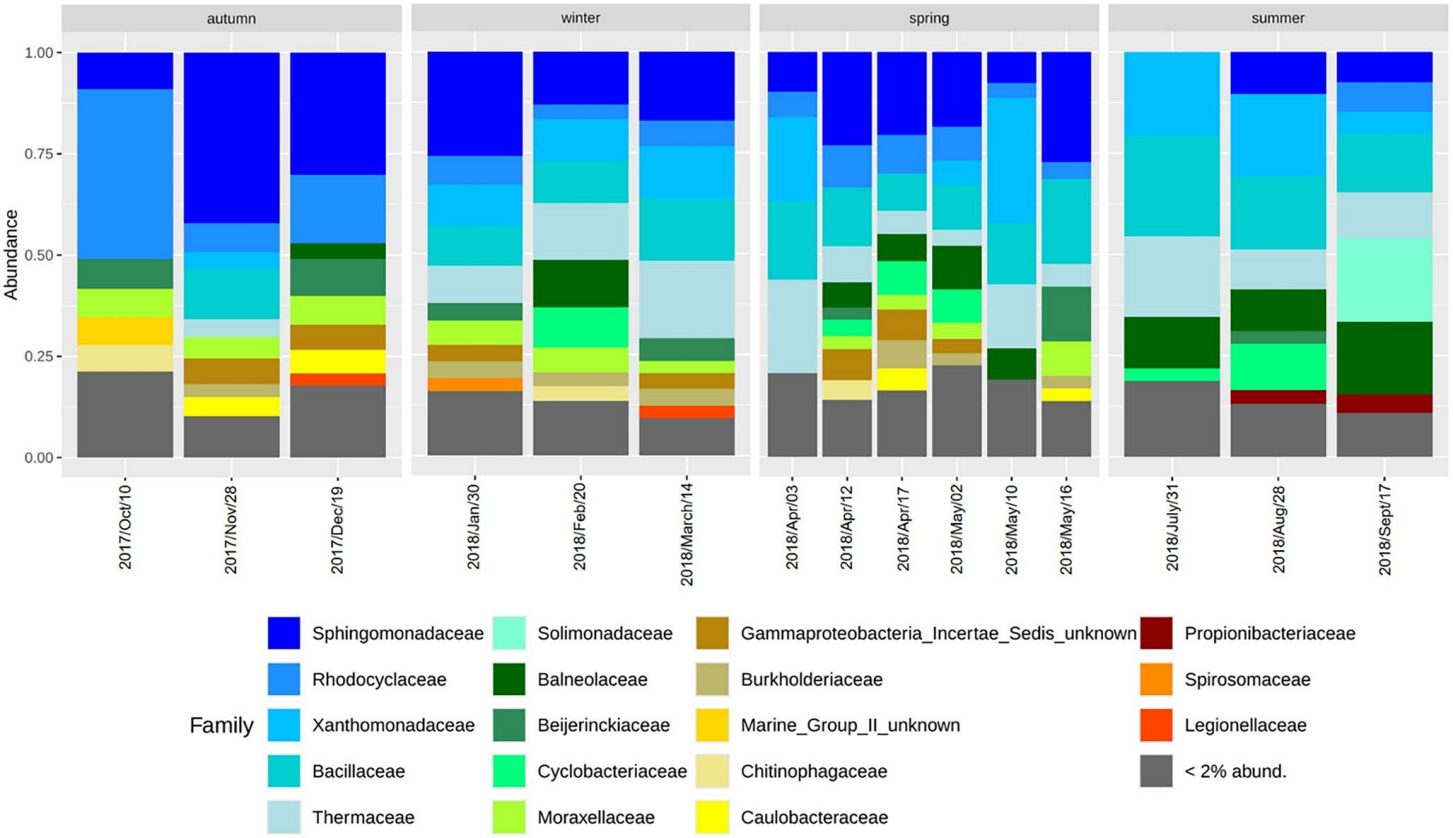
Relative abundances of prokaryotic families. The families with abundance < 2% in each sample are grouped and represented as ‘< 2% abund.’, in grey.

At the family level, the most abundant components were Sphingomonadaceae (Alphaproteobacteria), Bacillaceae (Firmicutes), Thermaceae (Deinococcus‐Thermus), Xanthomonadaceae and Rhodocyclaceae (Gammaproteobacteria), with 17%, 13%, 10%, 10% and 9% of total ASVs, respectively. The Sphingomonadaceae were always the most abundant family in all seasons except during the summer when the most abundant was the Bacillaceae with 19% (Figure 4).

### 3.3 Bipartite Networks

**FIGURE 5:**
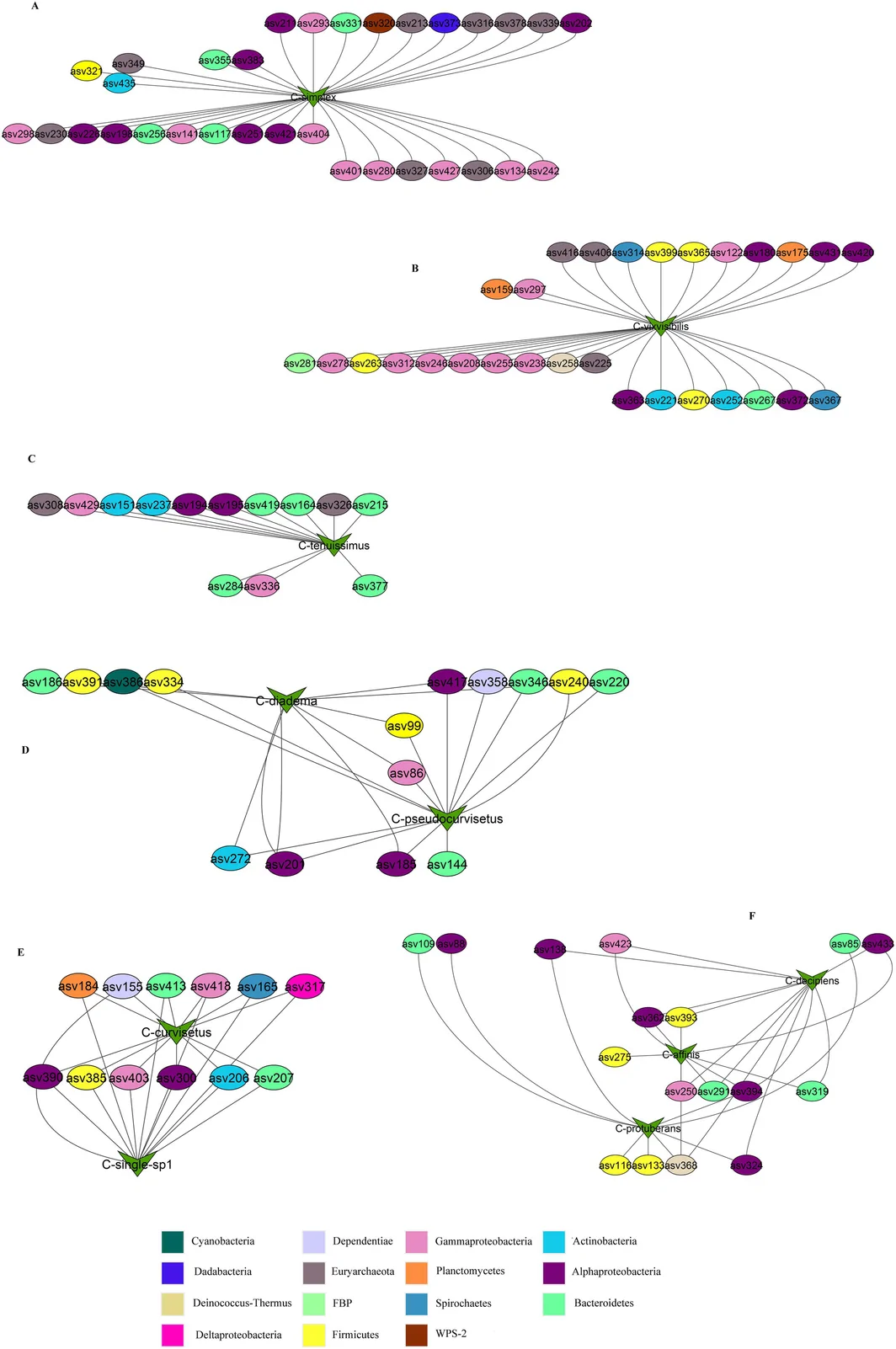
Bipartite networks. (A, B and C) show microbial associations involving a single *Chaetoceros* species. (D, E and F) show microbial associations involving multiple *Chaetoceros* species. *Chaetoceros* species are shown as dark green, while different prokaryotic ASVs are shown as ovals and colour‐coded at phylum level as in the legend.

After excluding ASVs with less than 5 reads, input tables included 442 prokaryotic ASVs and 20 *Chaetoceros* species. Network inference identified six distinct networks with 10 *Chaetoceros* species (50% of the total species) and 118 ASVs prokaryotic ASVs (27% of total ASVs) (Data S3). Three networks identified prokaryote ASVs associated exclusively with only one species of *Chaetoceros* ( *C. simplex* , *Chaetoceros vixvisibilis* and the colonial form of *C. tenuissimus* ) (Figure 5A–C). In the other three, prokaryotic ASVs shared connection with two ( *C. curvisetus* + *C. single‐sp1*; *C. pseudocurvisetus* + *C. diadema* ) or three species ( *C. protuberans* + *C. decipiens* + *Chaetoceros affinis* ) (Figure 5D–F).

**FIGURE 6:**
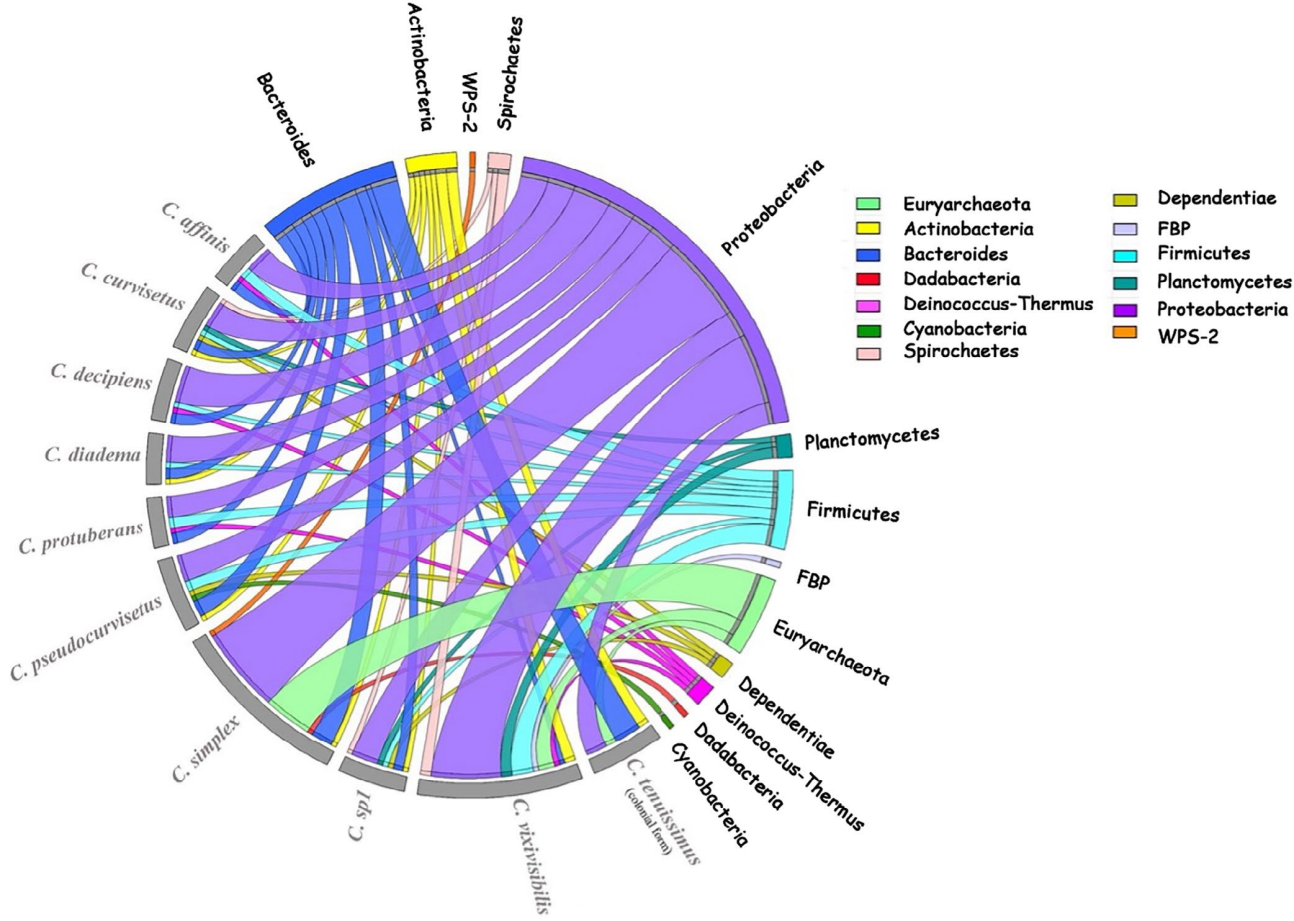
Links between *Chaetoceros* species (on the left in light grey) and prokaryotic phyla (on the right in black) selected by network inference. For each phylum the number of ribbons corresponds to the number of ASVs and the ribbon width is proportional to the abundance of the ASV.

All associations are visualised in Figure 6. Proteobacteria (mainly Alphaproteobacteria) and Bacteroidetes were the dominant phyla in the networks, being associated with all *Chaetoceros* species; Firmicutes co‐occurred with all species except *C. tenuissimus* and *C. simplex* , whereas Actinobacteria co‐occurred with all except *C. affinis* , *C. decipiens* and *C. protuberans*. Other phyla were represented in fewer networks. In particular, Deinococcus‐Thermus co‐occurred with only 4 *Chaetoceros* species ( *C. vixvisibilis* , *C. affinis* , *C. decipiens* and *C. protuberans* ); Spirochaetes and Planctomycetes co‐occurred with only 3 ( *C. curvisetus* , C. single_sp1 and *C. vixvisibilis* ); Euryarchaeota co‐occurred with other 3 ( *C. vixvisibilis* , *C. tenuissimus* and *C. simplex* ); so as Dependentiae ( *C. curvisetus* , C. single_sp1 and *C. pseudocurvisetus* ). Finally, some phyla included only one ASV connected with a specific *Chaetoceros* species: ASV of candidate‐phylum‐WPS was connected only with *C. simplex* ; ASV of Cyanobacteria was connected only with *C. pseudocurvisetus* ; ASV of candidate‐phylum‐FBP was connected with *C. vixvisibilis* and ASV of Dadabacteria was connected only with *C. simplex* (Data S4).

At the genus level, the network identified 48 distinct genera with a different number of ASVs. *Sphingomonas and Sphingobium*, *Methylobacterium* and *Brevundimonas* (Alphaproteobacteria) included the higher number of ASVs with 6, 4, 5, 4, respectively. Other genera were also well represented, such as *Dyadobacter* (four ASVs) and *Ekhidna* (four ASVs) belonging to Bacteroidetes class as well as *Deinococcus* (four ASVs) from Deinococcus‐Thermus phyla. Finally, Dadabacteria, Cyanobacteria, FBP and WPS‐2 included only one ASVs.

### 3.4 Relationship Between Prokaryotic Communities and Environmental Factors

Environmental parameters (temperature, salinity and chlorophyll; Table 1) were used in BIO‐ENV analysis to explore their relationship with prokaryotic community structure. For the total ASV dataset, temperature and salinity were identified as the environmental variables best associated with community structure, although the correlation was weak (Spearman's ρ = 0.093). In the subset of ASVs associated with *Chaetoceros*, salinity and chlorophyll emerged as the variables best associated with prokaryotic network structure, but again the correlation was weak (Spearman's ρ = 0.143).

**TABLE 1:** Environmental parameters collected at surface at the LTER‐MC station from October 2017 to September 2018.

Date	Temperature (°C)	Salinity (psu)	Chlorophyll (μg L^−1^)
10 October 2017	21.67	38.25	2.10
28 November 2017	17.93	38.23	0.97
19 December 2017	16.37	38.17	0.31
30 January 2018	14.69	37.92	3.38
20 February 2018	13.80	37.37	0.27
14 March 2018	14.58	38.14	0.71
3 April 2018	14.47	37.60	1.70
12 April 2018	16.24	37.44	3.41
17 April 2018	16.59	37.68	0.88
2 May 2018	19.74	37.30	1.90
10 May 2018	19.97	36.95	1.90
16 May 2018	18.66	37.44	0.87
31 July 2018	28.01	37.12	3.73
28 August 2018	26.39	37.81	0.64
17 September 2018	25.94	37.81	0.28

## 4 Discussion

Microalgae and their associated prokaryotes represent the basis of marine food webs, since they control the fate of carbon and the recycling of major elements. However, their relative dynamics are not simply passively determined by the fluctuation of seasonal and/or spatial abiotic conditions but result also from chemically mediated interactions (Ribalet et al. 2008; Cirri and Pohnert 2019). In this study, counts of *Chaetoceros* species were crossed with metabarcoding of particle‐attached (i.e., diatoms) prokaryotic communities, sampled over 1‐year time at a coastal site, to obtain statistically significant associations by bipartite network analyses. While it must be acknowledged that combining molecular data (16S metabarcoding) for prokaryotes with morphological data (LM) for diatoms introduces distinct methodological biases that must be carefully considered, we chose this dual approach based on the current standards and strengths of each respective field: for prokaryotes, HTS is necessary, as the vast majority of these taxa cannot be cultured or differentiated morphologically. Instead, for the diatoms, while the use of metabarcoding (e.g., using 18S or rbcL) is growing, light or electron microscopy remain the gold standard for traditional taxonomical assignment. Diatom morphological identification allows for direct calculation of established biotic indices, and avoids well‐known molecular biases such as differential DNA extraction efficiency, gene copy number variations and incomplete reference databases. Because our downstream analyses (such as BIO‐ENV) rely on nonparametric rank correlations of independent dissimilarity matrices (Bray‐Curtis), the structural pattern of each community is evaluated against the environment independently. Therefore, the methodological differences between the two biological groups do not mathematically artefact or compromise the environmental correlation results.

While temperature and salinity were the variables best associated with the total community, whereas salinity and chlorophyll were associated with the subset of ASVs involved in *Chaetoceros* networks, the low correlation coefficients suggest that additional factors, including biological interactions and host‐specific processes, may contribute substantially to the assembly of *Chaetoceros*‐associated prokaryotic communities. Interestingly, chlorophyll emerged among the variables associated with the network‐associated ASVs, whereas it was not retained for the total community. This suggests that phytoplankton dynamics may contribute to shaping prokaryotic assemblages associated with *Chaetoceros*, supporting the hypothesis that host‐related processes can influence microbiome recruitment within the phycosphere.

From a methodological point of view, the results allowed the identification of both new and previously known microalgae‐prokaryote associations, supporting the use of bipartite networks in this kind of studies. Indeed, while in unipartite global networks all nodes (species) are categorised equally, bipartite network analysis focuses on the interactions between nodes belonging to different guilds, in our case, *Chaetoceros* species versus prokaryotes. Taxonomic accuracy and data resolution are important, since species over‐splitting or aggregation, as well as low‐level taxonomy or wrong assignment, can be confounding factors. For this reason, the metabarcoding approach was adopted for the prokaryotes, as the vast majority of these taxa cannot be cultured or differentiated morphologically. However, despite all its benefits, limitations due to the size of the amplicon (300 bp) or missing reference sequences do not allow taxonomical resolution to reach the species level, limiting functional inference (Djemiel et al. 2022). Such limitation was evident in the prokaryotic dataset in which only 32% of the ASVs could be annotated as species. Yet, 89% found a match with environmental sequences (uncultured), allowing to identify their genera.

For the eukaryotes, the metabarcoding approach has been successfully applied to protists and diatoms communities in Gulf of Naples at LTER‐MC station in several ecological studies (Piredda et al. 2017; Ruggiero et al. 2022). However, the taxonomic profile used in this study for *Chaetoceros* genus was, instead, extracted from LM dataset regularly generated for marine plankton by expert taxonomist at LTER‐MC station. It is well known that LM can underestimate the species by aggregation of cryptic taxa, but similar biases could be also included in metabarcoding assessments with species over‐splitting and/or aggregation (intra species split populations or lack of discrimination at the species level). In addition, it must be considered that lab experiments focusing on microalgal‐bacteria interaction are based on morphological isolates from environmental samples. Thus, the use of LM data for *Chaetoceros* could offer the advantage to make strain selection easier in further laboratory or in situ studies.

In general, from a diatom perspective, our results support the possibility of a control mechanism by the host (Shibl et al. 2020), since several distinct associations and sets of bacterial ASVs for each *Chaetoceros* species, were observed. This is in line with studies on *Ditylum, Thalassiosira, Asterionella* diatoms (Schäfer et al. 2002), but in contrast with what observed in *Leptocylindrus* for which bacterial communities were quite conserved (Ajani et al. 2018).

Interestingly, most of *Chaetoceros* species involved in microbial associations are chain‐forming species (except for *C. simplex* and the not yet described *C*. sp.1), suggesting a role of this trait in supporting the association. However, colony‐forming *C. socialis* , one of the most common diatoms in the Gulf of Naples (Pelusi et al. 2020), was not included in any network, confirming its inability to be co‐cultured with prokaryotes, as previously reported (Deng et al. 2022). There are no supporting data to explain this feature, but it could be hypothesised that this species has defence mechanisms against possible colonisers. Chain formation is a common trait in diatoms, usually considered to be a trade‐off between resource acquisition and predator defence, and being beneficial in turbulent, high nutrient and predators conditions, although disadvantageous in low nutrient and nonturbulent conditions (Dell'Aquila et al. 2017; Kenitz et al. 2020). Chain formation as well as size are influenced by the combination of several factors as light, nutrient quality and quantity, turbulence and presence of grazers (Kenitz et al. 2020). Graff et al. (2011) reported that longer diatom chains have a higher probability of being colonised by prokaryotes and observed a selective bacterial attachment to specific regions of the chains. However, not all chains are the same, in fact, different strategies in dissolved inorganic nitrogen (DIN) assimilation are observed in *C. affinis* and *Skeletonema costatum* , with only the first being able to alleviate diffusion limitation through ‘microbial gardening’, that is, active selection of beneficial prokaryotes in the phycosphere (Stenow et al. 2023, 2024). In general, based on our results and the available literature, preferential associations between diatoms and fitness‐enhancing prokaryotes cannot be univocally proven, even though microbial transient ‘gardening’ could be a possible strategy to support chain‐forming diatoms when resource limitation occurs and as such it deserves further attention.

Studies on the recruitment and maintenance processes shaping diatom‐bacteria associations report conflicting evidence with respect to the relative importance of selective eco‐evolutionary dynamics vs. a neutral lottery‐based assembly mechanism, which might influence differently short‐ and long‐term association (Stock et al. 2022). Alphaproteobacteria and Bacteroidetes were the main phyla associated with our *Chaetoceros* species, confirming the results emerged from a comprehensive review of the current literature on diatom‐bacteria interactions (Hu et al. 2025) also suggesting, that components of these phyla hold specific traits favourable to attachment/interaction with diatoms. Unexpectedly, no ASVs were assigned to the *Roseobacter* clade, which has often been reported as associated with diatoms, both in culture and in nature, especially during blooms (Behringer et al. 2018; Hahnke et al. 2013).

From a prokaryotic perspective, when the lower taxonomical level was investigated (family to genus), selective interactions were evident. Two *Chaetoceros* assemblies showed putative associations with *Sphingobium* ( *C. simplex* , *C*. sp.1, *C. curvisetus* and *C. vixvisibilis* ) and *Sphingomonas* ( *C. diadema* , *C. pseudocurvisetus* , *C. affinis* , *C. decipiens* ) of Sphingomonadaceae family, which are both able to grow in the absence of organic substrates using polysaccharides produced by some diatoms (e.g., *Nitzschia* spp. and *Synedra acus* ) as a sole carbon source (Bruckner et al. 2008; Zakharova et al. 2010).

Also, the Alphaproteobacteria genera *Methylobacterium*, *Brevundimonas* and *Paracoccus*, present in this study, have been previously observed as associated with microalgae. *Methylobacterium* can grow on C1‐compounds as sole source of carbon and energy, which can also be exuded by algae (Krug et al. 2020) and it has been found to co‐occur with microalgae in natural biofilms (Krug et al. 2020), and as part of the phycosphere of several microalgae such as *Chlorella*, *Scenedesmus*, *Micrasterias* and *Chlamydomonas* (Levy et al. 2009; Calatrava et al. 2018). Zakharova et al. (2010) isolated *Brevundimonas* sp. from dead *Synedra acus* (a diatom), thriving on the frustules of decomposing algal cells hydrolyzing proteins, carbohydrates and other polymers. *Paracoccus* commonly co‐occurs in algal cultures or as epibiont of macro algae at sea, also showing resistance to polyunsaturated aldehydes and other toxic molecules released by diatoms (Amin et al. 2012; Crenn et al. 2018).

Members of Bacteroidetes, the second most abundant phylum in our networks, are usually reported to fast profit from lysing phytoplankton at transition areas, dominating phytoplankton bloom‐associated bacterial communities and usually involved in the initial degradation of blooms (Teeling et al. 2012; Buchan et al. 2014). An attached lifestyle has been observed for many diverse Bacteroidetes taxa that typically prefer to grow on algal surfaces and in the phycosphere of senescent phytoplankton (Amin et al. 2012; Buchan et al. 2014). Particularly during final stages of blooms at sea, Bacteroidetes are known to colonise particulate organic matter and switch from free‐living to particle‐attached lifestyle (Kirchman 2002; González et al. 2011). Other Bacteroidetes observed in this study included Flavobacteriales, Cyclobacteriaceae and *Sediminibacterium*, all previously reported also from sediments containing a demised diatom bloom (Smith et al. 2017).

Archaea were also found in our samples, associated with three *Chaetoceros* species ( *C. vixvisibilis* , *C. simplex* and *C. tenuissimus* ), possibly utilising and responding to dissolved organic matter and signalling molecules released by algae (Amin et al. 2012). Members of Thermoplasmata are the second most common archaeal group coexisting with microalgae and Marine Group II (MG‐II) has been reported as very abundant during a diatom bloom also containing *Chaetoceros* spp. (Needham and Fuhrman 2016).

In our networks, diazotrophic (N‐fixing) cyanobacteria were rare, with *C. pseudocurvisetus* associated with only one cyanobacterial ASVs assigned to the nitrogen‐fixing genus *Pleurocapsa*. This supports the hypothesis that diazotrophs are ruled out by the high availability of fixed inorganic N in coastal waters (Landolfi et al. 2015), even though several ecotypes of the symbiotic cyanobacterium UCYN‐A have been observed in coastal areas (Robicheau et al. 2023). Previous studies reported diazotroph bacteria of genus *Richelia* as endosymbionts of *Hemiaulus, Rhizosolenia* and *Bacteriastrum* diatoms, while *Calothrix* was found attached externally to *Chaetoceros* spp. (Tuo et al. 2021). Such association is beneficial resulting in higher levels N fixation in symbiont cyanobacteria than when grown alone (Foster et al. 2011).

Our study could not distinguish between synergistic and antagonistic interactions and some of the networks may also include passive aggregates of prokaryotes degrading dead phytoplankton within the so‐called detritosphere (Biddanda and Pomeroy 1988). Moreover, networks included prokaryotes often related to freshwater or terrestrial environments and most of these are reported to be potentially able to degrade several xenobiotics and other environmental contaminants and pollutants. *C. simplex* , for example, showed association with Dadabacteria, WPS‐2 phyla, four Rhodocyclaceae ASVs, *Sphingopyxis* and *Acidithiobacillaceae KCM‐B‐112* genera, which include taxa with a recognised role in carbon, nitrogen, phosphorus and sulphur recycling, probably related to the multiple anthropogenic activities located along the coast, in close vicinity to the highly polluted city of Naples.

Finally, we also found *Acidisoma* (Alphaproteobacteria) and *Anoxybacillus* (Bacillaceae), reported to be able to decompose cellulose and derivatives (Mieszkin et al. 2021), *Bacillus* sp., able to biodegrade herbicides (Durand et al. 2010), *Devosia* (Alphaproteobacteria), mainly reported in contaminated soils (Talwar et al. 2020), as well as the freshwater Gammaproteobacteria *Legionella*, pathogenic microorganism and Burkholderiaceae, that are common in soil and wastewater treatment systems, playing an important role in the degradation of organic pollution and denitrification of effluents (Huang et al. 2019; Cui et al. 2021). All these indicate terrestrial and freshwater inputs from the several sources located along the coast, including the Sarno river, one of the most polluted in Europe (Di Leo et al. 2020), whose inputs can be advected in the Gulf, depending on the circulation patterns.

## 5 Conclusions

Our study provides an overview of the prokaryotic members potentially involved in the phycosphere of *Chaetoceros* spp. in a marine coastal area spanning across seasons.

Although the interpretation of inferences by network analyses requires caution, our results highlight an active role of the host and the attached prokaryotes, suggesting that, based on the need of essential nutrients and cofactors, diatoms modulate associations possibly using specific secondary metabolites, recruiting several transient and beneficial bacterial assemblages in the phycosphere. Indeed, such modulations might preferentially occur in chain‐forming diatoms, especially in response to abiotic conditions or life stages. Based on the distinct networks obtained in our analysis, we can speculate a role of the host genotype in the recruitment from prokaryotic assemblages co‐occurring with diatoms in different geographic locations, environments or seasons. Alphaproteobacteria and Bacteroidetes are confirmed as the preferred phyla associated with diatoms, with also other prokaryotes associated with *Chaetoceros* spp. reflecting inputs from surrounding areas (e.g., terrestrial and freshwater). Further improvements in sequencing techniques will surely prove useful in refining taxonomical definition and in elucidation of functional guilds of associated prokaryotes (through metagenomics).

## Author Contributions

**Anna Chiara Trano:** writing – original draft, writing – review and editing, methodology, investigation, formal analysis, data curation, conceptualization. **Roberta Piredda:** writing – review and editing, supervision, methodology, data curation, conceptualization. **Raffaella Casotti:** writing – review and editing, conceptualization, supervision, project administration, funding acquisition.

## Funding

This work was supported by the European Commission (101000858) and the Stazione Zoologica Anton Dohrn.

## Ethics Statement

The species investigated (Bacillariophyceae, Bacteria and Archaea) are not regulated in EU member states according to Directive 2010/63/EU and therefore no ethical clearance is needed for this study.

## Conflicts of Interest

The authors declare no conflicts of interest.

## Supporting information


**Data S1:** Abundance (cell L‐1) of Chaetoceros species identified by Light Microscopy.

**Data S2:** Normalised dataset of prokaryotic ASVs with taxonomical assignment.

**Data S3:** List of Chaetoceros species and associated prokaryotic ASVs as inferred by CoNet software.

**Data S4:** Input table used in Circos software for the generation of the taxonomic overview in Figure 5.

## Data Availability

Sequencing data have been deposited in the National Center for Biotechnology Information (NCBI) Sequence Read Archive (SRA) under the BioProject ID: PRJNA1338595. Link to access the data: https://www.ncbi.nlm.nih.gov/sra/PRJNA1338595.
